# Insights into the Biochemical and Immune Mechanisms in Drug-Induced Liver Injury Pathogenesis

**DOI:** 10.1021/acs.chemrestox.5c00530

**Published:** 2026-02-12

**Authors:** Eleanor Saville, Georgia Wells, Liam Farrell, Dean John Naisbitt, Xiaoli Meng

**Affiliations:** Department of Pharmacology and Therapeutics, 4591 University of Liverpool, Liverpool L69 3GE, U.K.

## Abstract

DILI is the leading cause of drug failure in clinical trials and withdrawal from the market. Certain intrinsic mechanisms of injury have been characterized such as the direct cytotoxicity exerted by NAPQI, a reactive metabolite of acetaminophen. However, presentation of DILI is highly heterogeneous with several idiosyncratic presentations being observed in patients. Such manifestations are often linked to aberrant immune activation although the biochemical mechanisms directing such responses currently evade complete understanding. This review consolidates current literature findings into potential mechanisms of immune-mediated DILI as well as risk factors which may polarize both the liver itself and certain individuals toward a drug-reactive phenotype. Current theories implicate neoantigen formation as a result of the generation of drug–protein adducts by both parent drugs and reactive metabolites. Responses to such adducts can be restricted to the presence of certain HLA alleles though these associations are identified through epidemiological means rather than mechanistic investigations. Further, susceptibility to DILI can be linked to nuance in the T-cell responses to HLA displayed antigens where basal levels of effector molecules and inflammation as well as the presence of liver resident immune cells, such as natural killer T-cells, can augment drug-specific immune responses.

## Graphical abstract

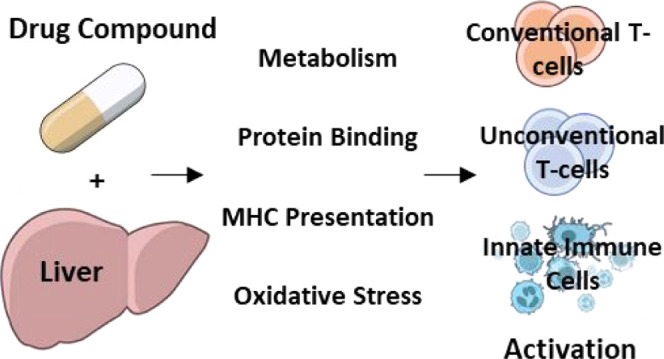

## Introduction

Drug-induced liver injury (DILI) manifests as a spectrum of clinical presentations such as hepatocellular injury caused by hepatocyte necrosis, and cholestatic patterns resulting from damage to the bile ducts.^1^ The overall mortality rate of DILI is around 10%; however, this figure can increase to up to 50% where acute liver failure has been induced.^2^ DILI is also the leading cause of drug withdrawal from the market as well as the principal cause of compound failure during clinical trials. Despite demonstrating a significant clinical risk and research burden, the heterogeneity of DILI manifestations and poorly understood pathomechanisms have stalled the deployment of therapeutic and diagnostics aids both in the clinic and in the drug development pipeline.

Intrinsic mechanisms of DILI have been somewhat delineated due to more pronounced links between the pharmacology of the drug and the pathology of the phenotype. The most well-characterized instances of intrinsic DILI are those associated with acetaminophen (APAP) where hepatotoxicity occurs as a direct result of the saturation of glucuronidation and sulfonation detoxification pathways directing the phase I metabolism of APAP toward the production of the toxic metabolite *N*-acetyl-*p*-benzo-quinone imine (NAPQI).^3^ In contrast, idiosyncratic DILI occurs independently of drug dose or duration and has less stringent associations with the action of the drug. Instead, immunological mechanisms are often implicated in cases not directly related to pharmacology and will be explored further throughout this review.

## Immune Involvement in DILI

Innate immune involvement has been identified in cases of intrinsic DILI whereby drug-injured hepatocytes release damage-associated molecular patterns (DAMPs) leading to the recruitment of tissue resident immune cells.^4^ The action of recruited immune cells including natural killer T (NKT) cells, neutrophils, and monocytes may then exacerbate injury through the release of inflammatory cytokines, chemokines, and reactive oxygen species (ROS).

Despite being more poorly understood than its intrinsic counterpart, an adaptive immune etiology is evidenced in the development of idiosyncratic DILI. For example, the time required for T-cell and B-cell expansion could explain the delayed onset often seen in idiosyncratic DILI cases with rapid recurrence of toxicity upon rechallenge of a drug indicating the presence of drug-specific memory cells. Indeed, histological analysis of drug-injured liver samples has revealed the presence of macrophages and infiltrating cytotoxic T-cells although their role in the establishment of DILI remains unclear.^5^ T-cell mediation is further implicated by the associations seen between drug-specific hepatotoxicity and certain human leukocyte antigen (HLA) alleles which are responsible for the presentation of antigens to T-cells. However, such restrictions are predominantly identified epidemiologically through genome wide association studies rather than mechanistic analysis thus limiting their significance in diagnostic applications. While effector immune responses in idiosyncratic DILI have been carefully observed, the biochemical pathways involved in the initiation of such responses are not as clearly defined and will be explored in this review.

## Formation of Reactive Drug Metabolites

Drug metabolism is a key process altering the chemical structure of drugs allowing for easier elimination from the body. This primarily occurs within the liver via three phases: I, II and III. Phase I metabolism, primarily but not exclusively mediated by the cytochrome (CYP) P450 enzymes usually involve oxidation and reduction pathways and are known to play a role in the onset of DILI with 60% of DILI-causing drugs metabolized by CYPs.^6^ Of these, CYP3A4/5 predominate metabolism, with CYP2C8/9 variants accounting for a quarter of metabolism when assessed using the Roussel Uclaf Causality Assessment Method.^7^

In the development of intrinsic DILI linked to APAP, metabolism via CYP2E1 is responsible for the formation of the hepatotoxic metabolite NAPQI.^8^ Although mechanisms of idiosyncratic DILI differ from intrinsic cases as they are independent of dose, the formation of toxic metabolites has also been seen to play a role in idiosyncratic DILI pathogenesis. An example of this is the CYP1A2 and 2C9 metabolism of naproxen to form 6-*O*-desmethylnaproxen which has been shown to stimulate CD4+ and CD8+ T-cells from NSAID DILI patients.^9^ Similar observations have been made for the anticonvulsant carbamazepine, with the CYP-mediated metabolite carbamazepine-10,11-epoxide shown to activate drug-specific T-cells.^10^ Furthermore, these processes may enhance the availability of reactive metabolites which form adducts with proteins thereby triggering a T-cell mediated immune response. In the case of the antibiotic dapsone, it undergoes CYP mediated oxidation to generate a hydroxylamine intermediate which spontaneously oxidizes to form nitroso dapsone which can covalently bind cellular proteins and prime T-cell responses in healthy donors.^11^ It has been demonstrated that the CYP3A4-mediated metabolites of nevirapine (primarily 12-hydroxynevirapine) are able to covalently bind to hepatic proteins, this has been proposed as the initiating event contributing to nevirapine-induced immune mediated liver injury, representing an archetype of CYP-dependent, metabolite driven DILI.^12^

Phase I metabolism is not, however, limited to CYPs with other oxidative drug metabolizing enzymes including monoamine oxidase and flavin-containing monooxygenase at play. These have been previously linked to drug hypersensitivity reactions, for instance in allopurinol reactions, the stable metabolite produced by xanthine oxidase and aldehyde oxidase enzymes is linked to the labile, pharmacological interaction with immunological receptors involved in eliciting T-cell responses.^13^

In contrast to their perceived detoxifying role, however, some of these metabolites have been observed to be involved in drug toxicity. Demonstrating this potential, an association between phase II reactive metabolite diclofenac acyl glucuronide accumulation and host genetic transporter variants affect patient susceptibility to diclofenac liver injury.^14^ O-acyl glucuronides have been the most widely studied of the phase II glucuronide metabolites in the context of drug toxicity. These are electrophilic glucuronides which can covalently modify proteins contributing to the induction of adverse drug reactions by hapten-induced activation of the immune system. Formation of these adducts have been well characterized through mass spectrometry and in vitro studies identifying that serum albumin is a major target of o-acyl glucuronides but many drugs are also deemed to target proteins located within the hepatocyte plasma membrane.^15^ Despite extensive studies on the covalent binding of acyl glucuronides to proteins, direct evidence that these reactive metabolites can trigger a T-cell response is still missing.^16–18^ Although nevirapine is extensively metabolized by phase I enzymes to hydroxylated metabolites, these intermediates may be subject to further phase II reactions, this includes glucuronidation but also sulfation and glutathione conjugation.^19–21^ Notably, the sulfated metabolite 12-sulphoxynevirapine remains chemically reactive and can form adducts with glutathione and cysteine residues.^21^ This highlights that some phase II products may themselves be reactive, forming protein adducts that contribute to hepatotoxicity. Overall, there exists a fine balance between detoxification (efficient conjugation and elimination) with bioactivation (reactive intermediate formation, insufficient detoxification and adduct formation). This is illustrated by nevirapine, where liver models show upregulation of phase II enzymes as an attempted detoxification response yet concurrent glutathione depletion may compromise this capacity, tipping the balance toward toxicity.^19^

Finally, Phase III relates to the transporter-mediated elimination of drugs or metabolites from the liver. Two superfamilies, the solute carrier (SLC) and ATP-binding cassette (ABC), mediate transport of substrates within the liver. Primarily, SLCs facilitate substrate influx but may also act as efflux transporters, while ABC transporters fulfill an efflux role. A role of the transporter proteins has been previously indicated within DILI with influx transporter levels reduced and export transporters increased in animal models on exposure to hepatotoxic compounds.^22^ While this may be suggestive of a protective capacity within the liver to regulate transporter expression to prevent accumulation of toxins, it has become increasingly apparent that drugs and their metabolites may also inhibit transporters responsible for the movement of biliary components; disruption of these systems results in intracellular accumulation of compounds. Troglitazone, bosentan and tolvaptan are alike in that their metabolic pathways result in metabolites that are inhibitors of the ABCB11 transporter protein within liver cells.^23^ Such transporter inhibition results in bile acid accumulation often underlying a cholestatic or mixed pattern of DILI, this may also sensitize hepatocytes to immune activation. Flucloxacillin can form reactive metabolites that covalently bind hepatocellular proteins.^24^ Blockade of efflux transporters such as MRP2 and *P*-glycoprotein reduces this covalent binding, suggesting that phase III transport modulates intracellular exposure to reactive intermediates and that impaired export may increase risk. While formation of these adducts and their transporter-dependent modulation alone do not guarantee DILI in vivo, it is plausible that protein adduct formation, in combination with transporter dynamics and host genetic susceptibility, provides a mechanistic pathway linking conjugation to immune-mediated liver injury.

With all this considered, it is clear there exists a complex interplay between the metabolism of a drug and its ability to potentiate the immune system. Consistent through phase I and II metabolism is that drugs and their metabolites are capable of producing ROS which can directly lead to damage of hepatocytes and other cells within the liver. Additionally, metabolism-formed compounds may be capable of conjugating with self-proteins able to interact with immunological receptors mediating T-cell immunity, detailed below.

### Neoantigen Formation and Presentation

HLA is used to describe the human specific set of genes encoding major histocompatibility complex (MHC) molecules whose function is to display peptides to T-cells. Under normal conditions, self-peptides for which T-cells have tolerance are displayed and no response is initiated. However, in cases of viral infection, pathogenic peptides are instead displayed leading to T-cell activation. It is known that drugs and drug fragments bound to self-peptides are also capable of elucidating aberrant immune provocation following display on MHC.^25,26^ Where drugs activate the immune system through the formation of protein adducts they are referred to as haptens. The theory of haptenation was proposed in the 1930s when Landsteiner and Jacobs stated that low molecular weight compounds were too small to be recognized by the immune system and could instead form covalent interactions with protein carriers to generate complexes capable of presentation by HLA.^27^ Chemically inert drugs are more likely to directly interact with immunological receptors such as MHC and TCRs where they are responsible for inducing adverse immune reactions.

To date, drugs spanning multiple drug classes have demonstrable hapten action as they typically display electrophilic characteristics enabling interactions with nucleophilic protein moieties. For example, the antibiotic amoxicillin is the single most common culprit drug associated with DILI; it is also a well-defined hapten.^28^ The beta-lactam ring is highly reactive with several proteins. The abundance and high ligand binding capacity of human serum albumin (HSA) make it a frequent target for amoxicillin modification particularly at the amino acid groups on lysine chain residues.^29,30^ Mass spectrometric analysis has additionally confirmed this preferential binding activity as well as identified the presence of amoxicillin-albumin adducts in patients.^31,32^ The resulting amoxicillin-modified proteins can undergo normal processing and degradation, with the resulting drug-modified peptides being loaded onto HLA molecules for display to T-cells on the surface. A number of haptens have been strongly associated with the presence of specific HLA alleles establishing the genes as risk predictors for the development of DILI (Table 1). To expand, other beta-lactams have displayed hapten characteristics with the resulting drug hypersensitivity being restricted to individuals possessing a given HLA risk allele. Flucloxacillin-induced DILI is associated with the HLA-B*57:01 with a negative predictive value of up to 99% for example.^33^ In some cases, it is the metabolite of a drug which forms hapten-carrier complexes as is the case with the nitroso metabolite of dapsone and the epoxide metabolite of carbamazepine. Additionally, adverse drug reactions to both of these compounds have been shown to be strongly restricted to the HLA class I alleles HLA-B*13:01 and HLA-B*15:02 respectively.^34,35^

**Table 1:** Drugs Associated with Immune-Mediated Liver Injury^a^ ^,^ ^b^

Drug	Antigen specificity	HLA associations	Drug-specific T-cells	Mechanisms of T-cell activation	Evidence of covalent binding	Ref
Amoxicillin	Amoxicillin	HLA-A*02:01, HLA-DRB1*15:01	CD4 > CD8	PI & Hapten	HSA	^26,42,64–66^
Flucloxacillin	Flucloxacillin	HLA-B*57:01	CD8 > CD4	PI & Hapten	HSA, hepatocellular proteins	^24,43,67^
Naproxen	6-*O*-desmethyl naproxen	Not known	CD4 > CD8	PI	Acyl glucuronides bind to HSA	^9^
Dapsone	Nitroso metabolite and dapsone	HLA-B*13:01	CD8	PI & Hapten	hemoglobin, HSA and cellular proteins	^68,69^
Sulfamethoxazole	Nitroso metabolite and sulfamethoxazole	HLA-B*38:01	CD4 > CD8	PI & Hapten	HSA, GSTP and cellular proteins	^10,70^
Carbamazepine	Carbamazepine & 10,11-epoxide	HLA-A*31:01HLA-B*15:02	CD8	PI	HSA, GSTP	^71,72^
Isoniazid	Isoniazid	Not known	CD4 > CD8	PI	HSA	^73–75^
Lumiracoxib	Not known	HLA-DQA1*02:01	ND	Not known	GSH adducts	^76,77^
Lapatinib	Not known	HLA-DQA1*02:01	ND	Not known	Quinone imine metabolite binding to GSTP & HSA	^78,79^
Terbinafine	Not known	HLA-A*33:01	ND	Not known	Aldehyde binding to GSTP	^80^
Tolvaptan	Tolvaptan, DM-4107, DM-4103	Multiple alleles	CD4 > CD8	PI	ND	^81,82^
Atabecestat	DIAT	Multiple alleles	CD4 > CD8	PI	GSTP & GSTA	^83–85^
Ticlopidine	Ticlopidine	HLA-A*33:03	CD8 > CD4	PI	GSH adducts	^86,87^
Ximelagatran	Not known	HLA-DRB1*07, HLA-DQA1*02	Not known	Not known	----	^88^

Biological Therapeutics					
Class	Example drugs	HLA associations	Drug-specific T-cells	Mechanisms of adverse drug reactions	Ref
Anti-TNF-α	Infliximab, adalimumab, etanercept	HLA-B*39:01	CD4 > CD8	Immune deviation, immunogenicity, viral reactivation	^44,51–54,57^
Immune checkpoint inhibitor	Nivolumab, pembrolizumab, atezolizumab, ipilimumab	Not known	CD4 (autoimmune CD8 seen in liver histology)	Immune deviation, immunogenicity, viral reactivation	^59–62^
Anti-CD20	Rituximab	Not known	CD4 > CD8	Immune deviation, immunogenicity	^56^
Anti-IL-6	Tocilizumab	Not known	CD4	Immunosuppression, immunogenicity	^63^

a Abbreviations: HLA, human leukocyte antigen; HSA, human serum albumin; GSTP, glutathione-*S*-transferase P;, ND, not detected; PI, pharmacological interaction.

b Associated reactive metabolites, HLA associations, activated T-cells identified in in vitro studies, and the ability of parents and/or metabolites to form protein adducts are also shown.

#### HLA Associations

MHC molecules are broadly divided into class I (HLA-A, HLA-B, and HLA-C) molecules, which are found on the surface of all nucleated cells and display endogenous peptides, and class II molecules (HLA-DR, HLA-DP, and HLA-DQ) which are only present on the surface of professional antigen presenting cells (APCs) where they display exogenous antigens. While there is nuance in how the two classes display peptides for presentation, their function in the context of drug antigen presentation is largely comparable (Figure 1).

**Figure 1:**
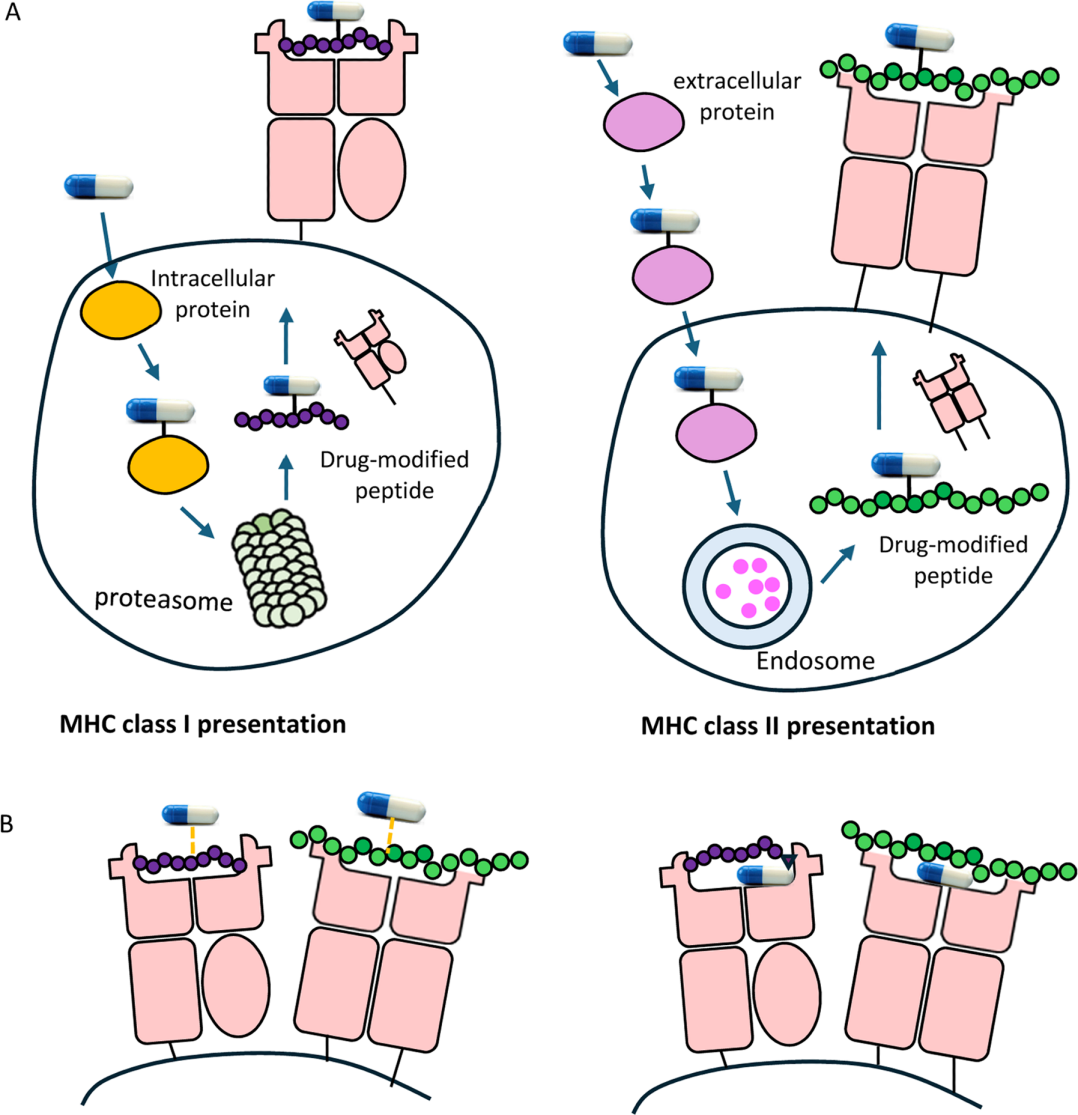
Drug-associated antigens can be presented by MHC molecules in an antigen processing dependent or independent pathway. Drug can form adducts with intracellular or extracellular proteins. Drug-modified proteins are processed to drug-peptide conjugates before display on the cell surface by MHC class I or class II molecules (A). Drug molecules can directly interact with peptides already presented on the cell surface or can alter peptide presentation through direct binding to MHC molecules (B).

Peptide binding grooves comprise of distinct pockets each displaying allele-determined preference for amino acids possessing certain biochemical properties due to variance in size, hydrophobicity, and electrostatic charge. For example, the binding pocket which interacts with the C-terminal of peptides displayed on HLA-A*02:01, the allele associated with amoxicillin-induced DILI, will preferentially interact with nonpolar aliphatic amino acid residues such as leucine and valine.^36^ As HLAs are the most polymorphic element of the human genome, an extensive variety of peptides can be displayed for T-cell scrutiny. Amino acid preferences at each binding site within the peptide-binding cleft of MHC contribute to the overall peptide repertoire of each HLA.

Some drugs can bind directly to HLA molecules and when the binding site is away from the peptide-binding groove, it may have little or no effect on ligand binding. However, if a compound binds within the binding groove, its position can influence the chemistry of peptide binding and potentially alter the repertoire of peptides presented by a given HLA allele. In particular, binding within the B or F pockets of the groove could shift the preference for peptide anchor residues, similar to how abacavir modifies peptide presentation by occupying the F pocket of HLA-B*57:01.^37,38^ Furthermore, drug modification on peptide side chains could influence their affinity for certain HLAs. Indeed, the influence of the position of the modified site on T-cell recognition of MHC displayed peptides must be considered but has so far not been investigated further than the discovery that the site modification within the peptide sequence was a key determinant of antigenic potential.^39^

Despite the negative predictive values of drug-HLA associations often being up to 99%, positive predictive values rarely rise above 10% indicating that a further restriction in addition to a specific HLA allele is required for T-cell activation to occur.^40,41^ Of course, as the generation of hapten-carrier complexes is dictated by the inherent chemistry of drugs and proteins, their formation and display would be expected in tolerant individuals as well as patients. Indeed, previous studies have confirmed the presence of hapten-carrier complexes in drug tolerant donors and T-cell responses can be invoked in non-hypersensitive healthy donors.^42,43^ An overview of example drugs known to elicit T-cell provocation through parent and/or metabolite protein adduct formation whose action may be restricted to specific HLA alleles is given in Table 1. This encompasses the well characterized small molecule compounds and new modalities such as biologics which are becoming increasingly clinically relevant although mechanisms of T-cell mediated hypersensitivity remain unclear.^44^ The use of biologics in the treatment of disease is growing rapidly, with a sizable share of the market at present and increasing numbers of candidates in development by pharmaceutical companies.^45,46^ Biologics are processed and presented to T-cells in a more straightforward manner than small molecules. Proteolytic degradation and subsequent presentation of linear peptide sequences lead to T-cell activation or tolerogenic mechanisms occurring.^47^ Non-native peptide sequences (or non-native structural elements) derived from such proteins are a key source of neoantigens to which the host is immunologically naïve alongside peptides that mimic existing epitopes to which the patient has preexisting immunological memory.^48,49^ Immunogenicity to biologics often refers to the generation of antidrug antibodies (ADAs), leading to direct toxicities or loss of efficacy of a given therapeutic due to neutralization. Generation of ADAs is common and often reliant on biologic derived-peptide specific CD4 T-cells to support these reactions.^50^ While rare, several biologics have been linked with cases of immune mediated liver injury. Although direct immunogenicity may be implicated in DILI pathogenesis, this is rare and largely limited to TNF-α inhibitor therapies (e.g., infliximab, adalimumab, etanercept, certolizumab).^44,51–53^ Infliximab has a predicted HLA association giving credence to this direct T-cell toxicity although ADAs in the liver may also play a key role in liver injury.^54^ A large number of biologics target immune mediators to treat autoimmune and oncological indications, meaning the pharmacology of the therapeutic must also be considered in treatment emergent adverse events. Furthermore, two additional mechanisms of liver injury are seen in treatment with immune targeting biologics: immunosuppression and immune deviation.^55^ Immunosuppression can lead to the reactivation of viruses such as hepatitis B and subsequent associated liver injury. Hepatitis B reactivation is commonly observed in immunomodulatory biologics such as TNF antagonists and anti-CD20 (e.g., rituximab) treatment.^56,57^ Immune deviation can lead to altered immune tolerance and homeostasis of T-cell subtypes, ultimately leading to autoimmune hepatitis.^57,58^ Treatment with all immune targeting biologics can lead to autoimmune hepatitis, immune checkpoint inhibitor antibodies (e.g., anti-PD-1: nivolumab, pembrolizumab; anti-PD-L1: atezolizumab; anti-CTLA4: ipilimumab) are however most commonly associated with cases of autoimmune hepatitis due to unregulated effector T-cell activation.^59–62^ Anti-IL-6 therapies (e.g., tocilizumab) have also been associated with hepatocellular liver injury, reportedly due to the reduction in liver repair mechanisms mediated by IL-6 in healthy individuals.^63^ DILI in treatment with biologics is not well-defined, further work is required to underpin the mechanisms of these reactions given the rapid evolution of biologics used in the treatment of disease. A summary of known DILI examples with the administration of biologics is seen in Table 1.

### T-Cell Mediated DILI

The role of T-cells in the development of idiosyncratic DILI is underscored by the frequent observation of T-cell infiltration in patients. One study quantified lymphocyte populations and surface markers in patients with DILI compared with non drug-induced liver injury controls, demonstrating increased numbers of activated CD4^+^ and CD8^+^ T-cells in peripheral blood during the acute phase of DILI.^89^ More directly, intrahepatic infiltration of CD8^+^ T-cells has been demonstrated in liver biopsies from patients with DILI, with the extent of infiltration correlating with clinical markers of liver injury.^40^ In the specific case of flucloxacillin-induced liver injury, patients carrying the HLA-B*57:01 allele exhibit hepatic infiltration of cytotoxic CD8^+^ T-cells, supporting a direct immune-mediated cytotoxic mechanism.^90^ Similarly, amoxicillin-clavulanate associated DILI cases frequently demonstrate portal and lobular infiltration by T-cells.^91^ In many cases, both parent drugs and their metabolites can contribute to T-cell activation through two main mechanisms: direct noncovalent interactions with the MHC-peptide complex (the pharmacological interaction, or the PI pathway), or covalent binding to cellular proteins followed by presentation of drug-peptide conjugates (the hapten pathway). For example, naproxen, tolvaptan, and atabecestat, as well as their stable metabolites, can directly activate T cells via the PI pathway, whereas certain reactive drug metabolites can stimulate T cells through the hapten mechanism. Importantly, these metabolites are generated in the liver and therefore have the potential to activate resident hepatic T-cells, potentially contributing to liver injury (Table 1).

Although many instances of T-cell mediated carbamazepine hypersensitivity manifest in the skin in a strongly HLA-restricted manner, hepatic pathologies have been found to accompany these presentations including instances of acute liver failure which have proved fatal in at least one case.^92^ Likewise, sulfamethoxazole has been shown to stimulate T-cells, predominantly via its oxidative metabolites, with one study describing how treatment resulted in hepatocellular injury in a patient with cystic fibrosis.^93^ In many cases, an inflammatory cytokine environment, such as that present in cystic fibrosis patients, can contribute to reaction potential with in vitro studies confirming that treatment of human hepatocytes with flucloxacillin or nitroso-sulfamethoxazole increases T-cell stimulation by triggering the release of TNF-α, IL-1, and IL-6.^94^

Chemokines within the liver create a microenvironment that facilitates T-cell homing and retention. Inflammatory chemokines such as CCL25, CCL21, CXCL9–11, CXCL16 and CCL4 have been shown to be particularly relevant in hepatic inflammation.^95^ In the context of DILI, this has been demonstrated functionally through expression of the chemokine receptors including CCR2, CCR4, CCR9 and CXCR3 on flucloxacillin-specific CD8^+^ T-cell clones.^67^ These have been widely regarded as receptors involved in the migration and accumulation of immune cells in the liver.^96,97^ A study generating T-cell clones from amoxicillin-clavulanate DILI patients similarly shows CD4^+^ and CD8^+^ clones expressing CCR4, CCR9 and CXCR3.^65^

Although the mechanistic basis of HLA-restriction in T-cell mediated adverse drug reactions is still unclear, the action of cytotoxic T-lymphocytes (CTLs) may extend beyond strict antigen specificity. Several mechanisms of cytotoxicity are exacted by CTLs including perforin and granzyme B release which induce cell death through direct cell lysis and the activation of apoptotic pathways following caspase activation. Additionally, cell surface expression of the Fas ligand (FasL), which binds the Fas death receptor on target cells triggering a downstream caspase cascade leading to apoptosis, is associated with CTL-mediated tissue damage.^98^ Interestingly, the Fas pathway is thought to exert nonspecific cell death as well as the targeted killing of APCs. In the context of DILI this would include the killing of cells which are not displaying drug antigens whether on the HLA allele associated with the culprit drug or otherwise. Indeed, such bystander killing has been observed in hepatocytes where HLA-B*57:01 restricted flucloxacillin-reactive T-cells kill both HLA-transduced and non-HLA-transduced liver cells.^90^ The high Fas expression seen in the liver may therefore be a factor in the sensitivity of the organ to T-cell mediated toxicity; this effect may be further compounded in individuals with existing liver pathologies where basal levels of Fas are elevated.^99^

Together, these findings indicate a pathogenic role for T-cells in DILI, alongside the identification of HLA risk alleles as previously mentioned, this supports a model in which drug or metabolite modified self-proteins are presented by HLA molecules to activate T-cells, driving the immune-mediated injury characteristic of idiosyncratic DILI.

### Involvement of Innate Immunity

Although the adaptive immune system has been the most well characterized contributor to the development of idiosyncratic DILI, the involvement of the innate immune system should also be considered. The principal innate immune cell populations within the liver include Kupffer cells, leukocytes and natural killer (NK) cells. Unlike the adaptive response, these pathways are not dependent on HLA restriction or TCR specificity. NK cells have been implicated in DILI through evidence of increased activation in mouse models of halothane-induced hepatotoxicity, additionally NK-derived IFN-γ has been shown to contribute to hepatocyte cytotoxicity and amplifies inflammatory damage.^100,101^ Kupffer cells and neutrophils also participate in innate inflammatory amplification, with neutrophil infiltration shown to exacerbate liver injury in triptolide-treated mice.^4^ Eosinophils have additionally been associated with DILI caused by several drugs, including acetaminophen, diclofenac, carbamazepine, enalapril and halothane, where their recruitment often reflects an allergic or hypersensitive response.^4^ Collectively, these findings indicate that DILI involves a multifaceted immune response, in which innate activation and cytokine production contribute to hepatocyte injury and help shape the subsequent adaptive immune response.

As discussed throughout this review, drug metabolism can see the formation of reactive metabolites or drug–protein conjugates which induce hepatocyte damage. Neutrophils and macrophages within the liver are believed to express CYPs, rendering them capable of generating reactive metabolites themselves.^102^ In response hepatocytes release DAMPs such as high-mobility group box 1 (HMGB-1), mitochondrial and nuclear DNA, and heat shock proteins which stimulate the innate compartment of the immune system and in turn further damages the hepatocytes and stimulates the adaptive immune response.^103^ Some of the key innate players and their roles in the pathogenesis of DILI are summarized in Table 2.

**Table 2:** A Summary Table of the Key Innate Immune Cells, Their Abundance within the Liver and Roles in the Pathogenesis of DILI^a^

cell type	liver abundance	roles in DILI	ref
Kupffer Cell	15% Total Liver Cells	Liver-resident macrophages	^103^
		Produce pro-inflammatory cytokines (IL-6, TNF-α) and ROS	
Natural killer (NK) Cells	Up to 50% intrahepatic lymphocytes	Produce cytolytic granzymes and perforin	^103^
		TNF-α and IFN-γ production	
Neutrophils	Low presence	Promote oxidative stress, mitochondrial dysfunction and necrosis	^104^
Eosinophils	Low presence	Degranulation of major basic protein and eosinophil peroxidase	^105^
		Type 2 cytokine production(IL-4, IL-13)	
Mast Cells	Low presence	Degranulation—release of histamines and TNF	^103^
		Stimulate hepatic stellate, Kupffer and adaptive immune cells	
Dendritic cells	Relatively rare	Main antigen presenting cells	^4^
		Produce cytokines (IFN-γ, IL-6 and TNF-α)	
		Promote activation of T-cells	

a Abbreviations: IFN, interferon; NK, natural killer; ROS, reactive oxygen species; TNF, tumor necrosis factor.

The conventional CD4^+^ and CD8^+^ T-cells that recognize peptides presented by MHC class I and II molecules are the best studied subsets in adaptive immunity. However, an additional group of ‘unconventional’ T-cells have emerged as a major component of innate-like immune surveillance, displaying greater abundance and immunological influence than previously understood. Unconventional T-cell subsets constitute a diverse population of lymphocytes specialized for rapid, innate-like responses. Unlike conventional T-cells, their antigen recognition is not restricted to classical MHC molecules. Instead, they employ distinct TCR conformations, typically semi-invariant αβ or γδ TCRs which recognize nonpolymorphic ligands.^106,107^ These ligands may be encoded within the MHC locus (e.g., HLA-E, HFE) or outside it, such as members of the Cluster of differentiation 1 (CD1) family, which presents lipid antigens and MHC class I related protein (MR1), which presents small molecule metabolites.

Based on their TCR usage and ligand restriction, unconventional T cells can be broadly divided into three groups: (i) semi-invariant populations such as mucosal associated invariant T (MAIT) cells and type I invariant natural killer T-(iNKT) cells, (ii) diverse populations including H2-M3– and HLA-E–restricted cells, and (iii) diverse CD1- and MR1-restricted cells such as type II NKT and γδ T-cells.^106^ Each group employs distinct mechanisms of antigen recognition reflecting their unlike TCR structures, which in turn shape their effector functions and developmental pathways. Due to their semi-invariant TCR usage, many of the unconventional subsets show highly conserved gene rearrangements and limited V­(D)­J diversity. This constrained repertoire is evolutionarily conserved across species underscoring their fundamental role in immune homeostasis.^108^ Among these innate-like subsets, MAIT, iNKT and γδ T-cells are highly enriched within the liver, where they exert both protective and pathogenic functions during inflammation and tissue injury (Figure 2).^109^

**Figure 2:**
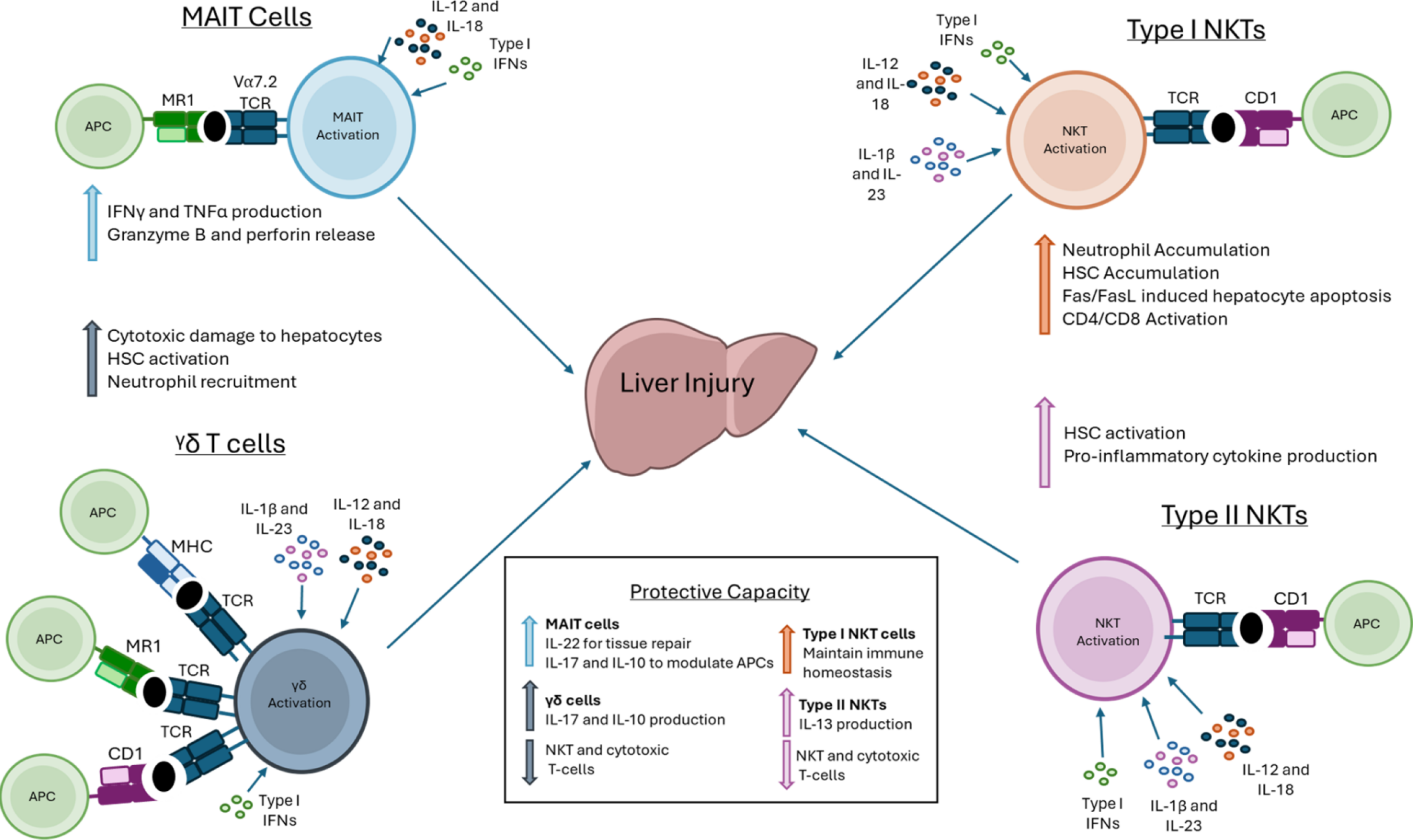
Summary of potential involvement of unconventional T-cells in liver injury. Antigen presenting cells (APCs) activate distinct subsets of unconventional T-cells, including mucosal associated invariant T (MAIT) cells, γδ T-cells and natural killer T-cells (NKT) (type I and II) through presentation of specific antigens or metabolites. Upon activation, cells including activated T lymphocytes, Kupffer cells, and innate-like T cells secrete cytokines that can influence the outcome of liver injury. Their cytokine and cellular interactions can either confer protection, by promoting repair and immune regulation, or exacerbate pathology by amplifying cytotoxic and inflammatory pathways leading to liver damage, which could be relevant to drug-induced liver injury (DILI) pathologies. Abbreviations: APC, antigen presenting cell; CD1, cluster of differentiation 1; MHC, major histocompatibility complex; MR1, major histocompatibility complex I-related protein; NKT, natural killer T-cell: MAIT, mucosal associated invariant T-cell, HSC, hepatic stellate cell; TCR, T-cell receptor.

Although unconventional T-cell subsets comprise only a small fraction (up to 10%) of circulating T-cells, they often represent a dominant population within tissues such as the liver.^110^ Their enrichment at this immunologically unique site, coupled with their ability to respond rapidly to stress-induced signals, lipid and metabolite antigens or drug-modified self-antigens positions them as key sensors of hepatocellular perturbations. Given this, unconventional T-cell subsets represent a critical but underexplored component of idiosyncratic DILI pathogenesis. Investigating their capacity to recognize drug-derived ligands may reveal early events in immune activation and explain interindividual susceptibility to adverse responses. These insights could ultimately help identify predictive immunological signatures of DILI. Therefore, the following sections will examine the contribution of unconventional T-cell subsets to liver injury and assess the evidence for their specific activation by drug antigens in DILI.

#### Natural Killer T Cells

NKT cells represent a T-cell population restricted by CD1d and specialized for recognizing lipid-based antigens. They express αβ TCRs alongside natural killer (NK) cell markers such as CD16.^111^ Two principal subsets of NKT cells exist: type I or (invariant) NKT cells, defined by an invariant TCRα chain paired with limited TCRβ variability, and type II (diverse) NKT cells, which exhibit a broader TCR repertoire. These subsets have opposing functional roles, with type I NKT cells generally promoting inflammation and type II NKT cells exerting anti-inflammatory effects.^112^

Type I NKT cells have been shown to rapidly accumulate in liver sinusoids within minutes of exposure to the synthetic glycolipid α-galactosylceramide, illustrating their capacity for swift intrahepatic activation.^113^ Following activation, they predominantly secrete IFN-γ but can also produce a spectrum of TH1-, TH2, and TH17-like cytokines depending on APC context.

To date, there is no clear evidence that drugs are directly presented by CD1 molecules in a manner analogous to peptide presentation by classical MHC, and no cases of CD1-mediated drug hypersensitivity have been described, Nevertheless lipid-based drug delivery systems could theoretically engage CD1-dependent pathways, and further research remains necessary to determine whether it is possible that drugs may modify endogenous lipids to generate neoantigens capable of activating NKT cells. Investigations of NKT cell involvement in DILI have yielded mixed outcomes. In APAP hepatoxicity models, NKT-deficient mice exhibit increased susceptibility to liver damage associated with elevated CYP2E1 expression, enhanced metabolic activation and greater APAP-protein adduct formation, suggesting a protective role for NKT cells.^114^ Conversely, in halothane- and triptolide-induced liver injury mouse models, depletion of CD1 or NKT cells, respectively, confers resistance to injury, indicating that NKT cells can also play a pathogenic role depending on the drug context.^115,116^

#### γδ T-Cells

γδ T-cells represent a distinct subset of innate-like lymphocytes that bridge adaptive and innate immunity. They express γ and δ TCR chains, which undergo a more restricted V­(D)­J recombination compared with conventional αβ T-cells. In humans, γδ T-cells constitute approximately 0.5–10% of circulating lymphocytes and 3–5% of liver lymphocytes, with their highest prevalence in the gut mucosa.^107^ Although γδ T-cells can recognize antigens presented by classical MHC complexes, they are best defined by their capability to detect antigens independently of MHC. They recognize a diverse range of nonpeptide ligands via molecules such as CD1, endothelial protein C receptor, MHC-related molecules (MICA/B) and MR1. These stress-induced molecules are typically upregulated on APCs during infection, transformation or cellular damage. In human peripheral blood, the predominant Vγ9 Vδ2 subset is activated by phosphoantigens produced by host cells and microbes, although the mechanism is incompletely understood, allosteric alterations of butyrophilin 3A1 are considered central to this process.^107^

γδ T cells are functionally versatile and highly shaped by their microenvironment. They can adopt Th1-, Th2-, Th9-, Th17- and regulatory-like profiles and secrete cytokines such as IFN-γ, TNF, IL-4, IL-9, IL-10, IL-17 and IL-22.^117^ Their capacity to respond rapidly without classical costimulation, often via a single activating ligand, positions them as first responders in tissue stress. In addition, γδ T-cells can act as APCs, modulating broader immune responses.^118^

Within the liver, γδ T cells include several effector populations which can contribute to both protective and pathogenic outcomes. Vδ1+ cells are highly enriched in hepatic tissues and are associated with cytotoxic activity, particularly in tumor models where they secrete IFN-γ.^119^ γδ T cells also produce IL-17 and IL-22 cytokines involved in tissue remodelling and fibrosis. IL-17-producing γδ T-cells can promote fibroblast proliferation and collagen deposition, implicating them in fibrotic liver disease.^119^

In DILI, γδ T cells appear to have primarily pro-inflammatory effects, especially through IL-17 production. In murine models of APAP-induced liver injury, DAMPs released from dying hepatocytes activate liver-resident γδ T-cells, leading to robust IL-17 secretion and subsequent neutrophil recruitment. Depletion of γδ T cells reduces hepatic IL-17A levels, neutrophil infiltration and overall liver damage, highlighting their pathogenic contribution in intrinsic DILI.^41^ The role of γδ T-cells in idiosyncratic DILI is however less well refined. Given their ability to respond to stress signals, lipids and metabolite-induced cellular changes, they may influence the disease through cytokine production, cytotoxic activity or modulation of other immune cells. However, direct evidence of γδ T-cell drug antigen recognition or drug-specific responses is currently limited.

Taken together, γδ T-cells function as rapid-response sentinels at the liver interface, capable of recognizing stress and xenobiotic perturbations. While they can contribute to tissue protection and immune surveillance, dysregulated γδ T-cell activation in DILI, particularly via IL-17 driven neutrophil recruitment, appears to exacerbate inflammation and hepatic injury. Further research is needed to determine whether selective targeting of pathogenic γδ subsets can drive DILI without compromising their protective roles.

#### Mucosal Associated Invariant T-Cells

MAIT cells are a subset of T-cells defined by their semi-invariant αβ TCR, which recognizes antigens presented by the evolutionarily conserved MHC class I related molecule (MR1).^120^ MAIT cells express a canonical Vα7.2-Jα33/12/20 TCRα chain paired with a restricted range of TCRβ chains and are commonly identified by their high expression of the C-type lectin CD161. They constitute approximately 1–5% of circulating T-cells but are highly enriched at mucosal surfaces, within the lungs and particularly in the liver where they can comprise up to 50% of intrahepatic T-cells.

The primary physiological role of MAIT cells is in antimicrobial defense. MR1 presents intermediates from bacterial and fungal vitamin B synthesis pathways, most notably riboflavin (vitamin B2) and folic acid (vitamin B6) intermediates, allowing MAIT cells to detect microbial infection.^121^ Ligands derived from the folic acid pathway generally inhibit MAIT activation, whereas riboflavin-based intermediates potently activate MAIT cells. Of these, the derivative 5-(2-oxopropylideneamino)-6-d-ribitylaminouracil (5-OP-RU) is the most potent. The repertoire of MR1 ligands has since been expanded to include small molecule compounds and pharmacological agents. For example, diclofenac and several of its metabolites have been shown to bind MR1 and induce weak MAIT cell activation compared with 5-OP-RU.^122^ Conversely, compounds such as 3-formylsalicylic acid (an aspirin analogue) and the methotrexate derivative 2,4-diamino-6-formylpteridine can compete with agonistic ligands for the MR1 binding cleft, thereby inhibiting MAIT activation.^123^ With all this considered, being the only unconventional T-cell subset known to respond directly to drug antigens via their antigen presenting molecule, MAIT cells represent a plausible link between pharmacological response and immune-mediated drug hypersensitivity reactions.

Within the liver, MAIT cells are positioned at the interface of the innate and adaptive immune environments. They are highly responsive to inflammatory cytokines such as IL-12, IL-18 and type I interferons and can exert both protective and pathogenic effects. In hepatic disease, MAIT cells have thus far been implicated in control and clearance of hepatitis C virus and in the apoptosis of hepatocellular carcinoma cells.^124^ However, their activation can also contribute to disease pathogenesis. MAIT cells localize around bile ducts, and interactions between CD40L expressing MAIT cells and CD40 expressing biliary epithelial cells presenting bacterial MR1 ligands may promote Fas-dependent epithelial apoptosis.^124–126^ In contrast, within the hepatocellular carcinoma tumor microenvironment, MAIT cells show reduced cytolytic activity, expressing higher levels of immune checkpoint molecules and their infiltration correlating with poorer clinical outcomes.^127^

Given their abundance in the liver, responsiveness to both microbial and pharmacological MR1 ligands and capacity for cytokine-driven activation, MAIT cells could play a yet unknown role in the pathogenesis of DILI. It is plausible that drugs or their metabolites could directly engage MR1 to stimulate MAIT cells or modulate their activation, contributing to early immune responses in susceptible individuals. Conversely, their cytokine-mediated responses could amplify inflammation secondary to hepatocyte stress or injury. Further research is needed to determine whether MAIT cell activation by drug-derived ligands represents a mechanism within DILI, but their unique positioning and known pharmacological sensitivity make them an attractive target for investigation.

#### Oxidative Stress

Despite being categorized distinctly, there is overlap between intrinsically defined mechanisms of DILI and the otherwise idiosyncratic immune-mediated DILI. To use the previous example of APAP, the toxic metabolite NAPQI forms irreversible covalent interactions with proteins and DNA resulting in deleterious ROS formation and glutathione depletion.^128^ High levels of ROS overwhelm antioxidant pathways leading to mitochondrial damage and hepatocyte death which subsequently trigger immune activation and inflammation. Lipid peroxidation has additionally been identified as a cause of liver cell necrosis with reference to multiple drugs including APAP as well as the antiarrhythmic amiodarone. Here, lipid radicals are responsible for the breakdown of the polyunsaturated fatty acids present in lipid cell membranes leading to disruption in membrane potential and ion gradients necessary for cell survival.^129^

Both the innate and adaptive immune systems can be influenced by oxidative stress with ROS-induced DAMP release recruiting innate immune cells as well as evidence of T-cell and B-cell modulation by ROS production. Interestingly, both up-regulation and down-regulation of T-cell activity has been identified in response to ROS with overall dysregulation being thought to contribute to the autoimmune characteristics often present in cases of immune-mediated DILI. One study describes how mitochondrial ROS production is necessary for antigen-specific T-cell activation while another reports that ROS prevents the recognition of MHC-peptide complexes by T-cell receptors.^130,131^ These effects are mirrored in B-cells where evidence of loss of function due to dedifferentiation is observed as well as a requirement for sustained ROS production in the ongoing proliferation of activated B-cells.^132,133^ Thus, the role of oxidative stress in both the pathogenesis and as a consequence of immune-mediated DILI is difficult to determine.

### The Hepatic Immune Microenvironment

It is clear that one of the primary reasons the liver is especially susceptible to drug-induced toxicity is its role as the main site of drug metabolism and detoxification with the resulting generation of harmful metabolites having been outlined earlier in this review. Despite previously being considered an immunologically tolerant organ, it is becoming evident, as discussed in the “Neoantigen formation and presentation” section, that this assumption is flawed. As also detailed, resident immune cells from both the innate and adaptive arms of the immune system provide constant surveillance and clearance of threats with a general overview of their role in the development of DILI shown in Figure 3. Additionally, a basal level of inflammation is present in the liver to maintain homeostasis in response to exposure to dietary antigens as well as the detoxification and metabolism of xenobiotics, and both commensal and pathogenic microbes.^134^

**Figure 3:**
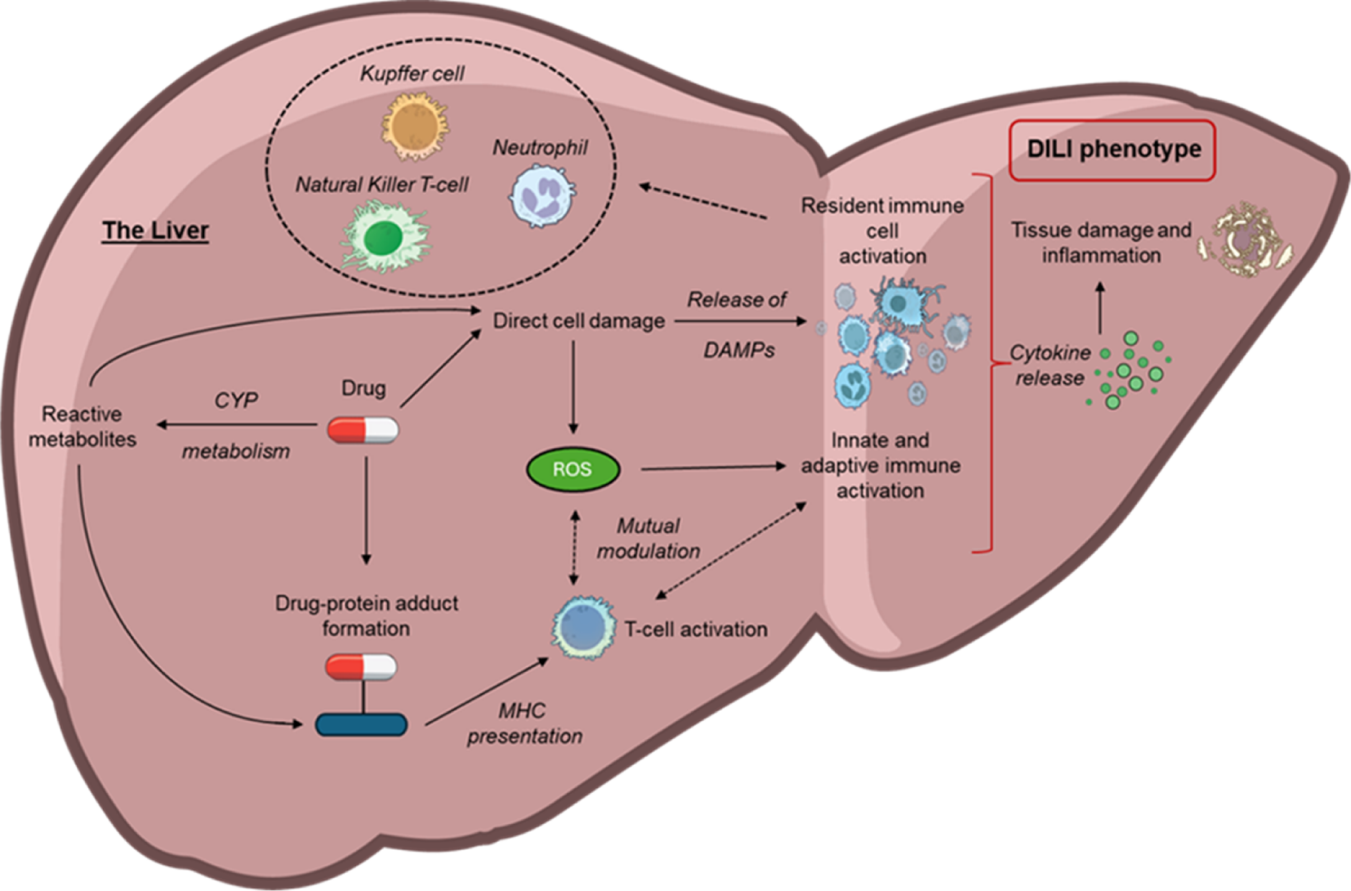
Summary of general mechanisms involved in immune-mediated drug-induced liver injury. Drug administration may cause direct cell damage leading to innate and adaptive immune activation in response to damage associated molecular patterns (DAMPs) with additional modulation by reactive oxygen species (ROS). Resident immune cells, including but not limited to Kupffer cells, neutrophils, and natural killer T-cells are also activated. Metabolism of drugs by cytochrome (CYP) enzymes can lead to the generation of reactive metabolites which may exert direct toxicity or form drug–protein adducts capable of stimulating T-cell directed immune responses. Aberrant immune activation then results in the release of cytotoxic cytokines, among other factors, leading to the cell death, tissue damage, and inflammation characteristic of the drug induced liver injury (DILI) phenotype.

This beneficial basal inflammation is highly regulated with failure to resolve inflammatory signals from gut microbes or toxic product clearance leading to autoimmunity through mechanisms as yet only theorized.^135^ Therefore, in such individuals where inflammation and the wider hepatic immune microenvironment is dysregulated and combined with the insult of drug metabolism and elimination, development of adverse immune reactions to drugs may be more likely. However, as the extent of liver resident immune cells and cytokine milieu remains undefined it is as yet unclear to what extent individual immune phenotypes contribute to the risk of DILI.

Of the cytokines involved in this milieu, tumor necrosis factor-alpha (TNF-α) and IFN-γ have been well indicated as essential to the pathogenesis of DILI.^136^ IFN-γ has been established to induce T-cell mediated hepatocyte death, inhibit proliferation of hepatocytes and regulate the activity of antigen-presenting immune cells.^137^ It is also believed to interact with TNF-α which plays a key role in liver pathophysiology and its contribution to DILI has been widely discussed. Its contribution in the context of intrinsic APAP-induced hepatotoxicity has been confirmed only in the presence of inflammation with APAP-treated transgenic mice only showing elevated TNF levels when additional inflammatory stimulus is present. Similarly, APAP-treated transgenic mice treated with anti-TNF antibodies are protected from liver injury but only in the presence of inflammation.^138,139^ A potentiating role of TNF-α has also been indicated in the development of idiosyncratic DILI caused by amiodarone and trovafloxacin.^140,141^ In further studies, TNF-α is identified as a key pro-inflammatory cytokine initiating drug-specific T-cell responses through lowering the activation threshold of naïve T-cells by downregulating V-type immunoglobulin domain-containing suppressor of T-cell activation (VISTA).^142^ This may pertain to be relevant in the context of idiosyncratic DILI in which TNF-α levels could dictate a loss in peripheral tolerance to drugs but this is yet to be explored. More work is therefore needed to establish how TNF-α alters T-cell responses to DILI drugs.

## Discussion and Future Perspectives

Immune-directed drug-induced hepatotoxicity is highly heterogeneous in presentation and multifaceted in pathogenesis. Factors relating to the pharmacokinetic processing of drugs and the environment in which this takes place can leave the liver vulnerable to deleterious immune activation with the effect compounded in certain patient populations.

Naturally, the role of the liver as the primary site of drug metabolism facilitates the production of metabolites able to induce injury both through direct tissue reactivity and the formation of neoantigens following host protein modification. However, as the intrinsic chemistry of the drug would remain consistent between patients, intra- and interindividual variables pertaining to drug metabolism should be investigated to determine if they influence generation of such immunogenic adducts. To expand, there is high population variability in the activity of drug metabolizing enzymes. For example, polymorphisms dictating expression levels of CYP3A4 can give rise to a population subtype of ‘poor’ metabolizers; such individuals retain higher systemic concentrations of drugs which can lead to a functional overdose display of toxicity.^143^ Whether such CYP variations could also direct the production of metabolites more likely to form neoantigens perhaps warrants investigation where allelic variants of CYP could be identified as susceptibility predictors similar to HLA risk alleles.

HLA screening has proven successful in reducing incidences of severe drug reactions, most commonly cutaneous conditions, however a lack of specificity makes the widespread adoption of such screening prohibitively inefficient.^144,145^ As drug-HLA restrictions are typically identified through genome-wide association studies, the determining mechanistic factors in such restrictions are unclear. Instead, investigating the variable regions of TCR sequences may reveal motifs required for drug recognition in patients experiencing adverse drug reactions and may provide a more robust metric for assessing risk of hypersensitivity.

Dynamic interactions between the innate and adaptive immune system are key to the pathogenesis of DILI, with reactive metabolites and drug–protein complexes known to initiate responses from both arms. A lack of in vitro models among a low incidence of DILI makes characterizing these interactions challenging. Current models rely on the co-culture of immune cells alongside target cells, but these largely focus on the conventional T-cell subsets. There is a need to expand these systems, particularly to incorporate innate-bridging T-cells which are seeing a rising role in liver disease progression, in order to appreciate the extent of immune cell involvement.

In addition to triggering both innate and adaptive activation, single drugs are known to induce distinct pathologies in different people. For example, amoxicillin is associated with both DILI and cutaneous manifestations separately as well as a mild self-resolving skin rash being the initial presentation of a more severe DILI phenotype in some patients.^146–148^ It remains unclear how a single antigen can elicit such varying presentations with further research into local and systemic immune populations being required to investigate how these heterogeneous reactions are directed which could provide further insight into possible pathology-specific treatment modalities. Furthermore, mechanistic understanding of the contribution of the unconventional T-cell subsets in the pathogenesis of DILI could provide new avenues for therapeutic intervention outside of risk stratification.

To summarize, immune involvement is weaved into multiple aspects of DILI with both the innate and adaptive arms able to mediate aberrant drug responses. Factors relating to the liver immune microenvironment and the role of the liver in drug metabolism may additionally make the organ especially susceptible to drug-induced injury. Genetic variations such as CYP expression and the presence of certain HLA alleles may additionally predispose individuals to a higher risk of DILI.

## Abbreviations

ABC: ATP-binding cassette
ADA: Antidrug antibodies
APAP: Acetaminophen
APC: Antigen presenting cell
CTL: Cytotoxic T-lymphocyte
CYP: Cytochrome
DAMP: Damage associated molecular pattern
DILI: Drug-induced liver injury
FasL: Fas-ligand
GST: Glutathione *S*-transferase
HLA: Human leukocyte antigen
HMGB1: High mobility group box 1
IFN-α: Interferon-gamma
MAIT: Mucosal-associated invariant T-cell
MHC: Major histocompatibility complex
NAPQI: *N*-acetyl-*p*-benzoquinone imine
NK: Natural killer
NKT: Natural killer T-cell
ROS: Reactive oxygen species
SLC: Solute carrier
TNF-α: Tumour necrosis factor-alpha
